# Integrative gene expression and heterologous functional analysis identify candidate regulators of apomixis in *Eragrostis curvula*

**DOI:** 10.3389/fpls.2026.1802327

**Published:** 2026-04-15

**Authors:** Ingrid Garbus, Juan Pablo Selva, Andrés Bellido, María Cielo Pasten, José Carballo, Cristian A. Gallo, Marta A. Mendes, Timothy F. Sharbel, Viviana C. Echenique

**Affiliations:** 1 Centro de Recursos Naturales Renovables de la Zona Semiárida (CERZOS), Universidad Nacional del Sur-Consejo Nacional de Investigaciones Científicas y Técnicas (UNS-CONICET), Bahía Blanca, Argentina; 2 Departamento de Agronomía, Universidad Nacional del Sur (UNS), Bahía Blanca, Argentina; 3 Dipartimento di Bioscienze, Università degli Studi di Milano, Milano, Italy; 4 Department of Plant Sciences, College of Agriculture and Bioresources, University of Saskatchewan, Saskatoon, SK, Canada

**Keywords:** apomixis, candidate gene identification, comparative gene expression analysis, *Eragrostis curvula*, reproductive development

## Abstract

**Introduction:**

*Eragrostis curvula* (weeping lovegrass) is a perennial forage grass in which diploid genotypes reproduce sexually whereas polyploids display pseudogamous diplosporous apomixis. Understanding the molecular basis of this reproductive system is important for the potential use of apomixis in crop improvement. Here, we applied a comparative gene expression analysis to identify and functionally assess candidate genes associated with apomictic reproduction in *E. curvula*.

**Methods:**

This analysis was performed using a custom 1×1M Agilent microarray designed from floral transcriptomes of sexual and apomictic genotypes. PCR screening across 14 genotypes and a segregating mapping population was conducted to evaluate candidate gene presence. Functional assessment was carried out through heterologous expression of the candidate genes in *Arabidopsis thaliana* using GoldenBraid 2.0 expression cassettes.

**Results:**

Of the 131 differentially hybridized probes identified, 130 were upregulated in apomictic plants. BLAST analyses revealed four main candidate genes, including a hypothetical protein (EcAPO1), a pre-mRNA splicing factor CWC22-like homolog (EcAPO2), a Cyclin-2A-1 (EcAPO3), and an F-box protein (EcAPO4). PCR screening showed that EcAPO1 and EcAPO2 were exclusively present in apomictic individuals, supporting their close association with the trait. Transgenic lines displayed abnormal floral and reproductive phenotypes, including homeotic transformations, supernumerary nuclei, embryo sac arrest, and altered endosperm development.

**Discussion:**

These results indicate that *E. curvula* candidate genes can disrupt conserved reproductive pathways and demonstrate biological activity in a heterologous system. Together, this integrative analysis combining comparative gene expression profiling and functional assays identifies novel candidate genes associated with apomixis in *E. curvula*. These findings provide a foundation for dissecting the genetic control of apomixis and advance efforts toward the applied manipulation of clonal seed reproduction in crops.

## 1 Introduction

Weeping lovegrass (*Eragrostis curvula* (Schrad.) Nees) is a perennial forage grass species native to southern Africa that has become widely naturalized in semi-arid regions worldwide, particularly in areas with sandy soils and frequent drought, where it shows high adaptability (Covas, 1991). This species constitutes a polymorphic complex with a basic chromosome number of x = 10, and ploidy levels ranging from diploid (2n = 2x = 20) to octoploid (8x = 80) (Bennett, 1972). While diploid individuals are rare and reproduce sexually, polyploid genotypes reproduce by apomixis (Voigt and Bashaw, 1976; Crane, 2001; Voigt et al., 2004).

Apomixis is defined as asexual seed formation that bypasses meiosis and fertilization, generating progeny genetically identical to the maternal plant (Barcaccia and Albertini, 2013). This reproductive strategy involves three key deviations from sexuality: the formation of unreduced female gametes (apomeiosis), embryo development without fertilization (parthenogenesis), and autonomous endosperm development or pseudogamy. The sporadic yet widespread occurrence of apomixis across unrelated angiosperm families suggests that this trait has evolved independently multiple times (Briggs and Walters, 2016).

In *E. curvula*, the female gametophyte develops through an Eragrostis-type diplosporous pathway, in which the megaspore mother cell undergoes mitosis rather than meiosis, giving rise to an unreduced four-nucleate embryo sac (ES) containing an egg cell, two synergids, and one polar nucleus (Meier et al., 2011; Carballo et al., 2021). Embryo development proceeds through parthenogenesis from the unreduced egg cell, while endosperm formation requires fertilization of the polar nuclei (pseudogamy), thereby maintaining the characteristic 2:3 (4:6) embryo-to-endosperm genomic ratio observed in sexual reproduction.

Furthermore, apomictic individuals may be obligate or facultative, in which sexual and apomictic ES are produced in variable proportions.

Although apomixis is absent from major crop species, its potential agricultural value is considerable. The capacity to generate clonal seed progeny would enable the fixation of heterosis and the rapid dissemination of elite genotypes, significantly reducing breeding time and costs associated with hybrid seed production (Schmidt, 2020; Underwood and Mercier, 2022). Consequently, substantial efforts have been devoted to elucidating the molecular basis of apomixis, both through the study of natural apomictic systems and by engineering synthetic apomixis-like mechanisms. These include the “Mitosis instead of Meiosis” (MiMe) system (D’Erfurth et al., 2009), combined with the induction of parthenogenesis via genes such as *BABY BOOM*-like (Boutilier et al., 2002; Conner et al., 2015; Khanday et al., 2019) or *PARTHENOGENESIS* (Underwood et al., 2022; Née and Romera-Branchat, 2022), as well as approaches involving modifications of *CENH3*, *MATRILINEAL*, or *DOMAIN MEMBRANE PROTEIN* genes (Kelliher et al., 2017; Yao et al., 2018; Zhong et al., 2019, Zhong et al., 2020). While the MiMe system has been successfully implemented in *Arabidopsis thaliana* (D’Erfurth et al., 2009) and in crop species such as rice (Wang et al., 2019; Khanday et al., 2019; Simon et al., 2025), its efficiency remains limited, highlighting the need to identify endogenous regulatory mechanisms operating in naturally apomictic species.

Comparative transcriptomic and genomic studies of apomictic plants relative to their sexual counterparts have been instrumental in identifying candidate genes and proposing regulatory mechanisms associated with reproductive mode determination (Ozias-Akins, 2006; Schmidt, 2020; Hojsgaard, 2020). In *E. curvula*, differential gene expression linked to reproductive development has been extensively investigated, leading to the generation of EST, cDNA, small RNA, and genomic sequence resources that have substantially advanced research in this system (Cervigni et al., 2008; Selva et al., 2012; Garbus et al., 2017, Garbus et al., 2019; Zappacosta et al., 2019; Selva et al., 2020). However, functional evidence connecting transcriptional variation with reproductive outcomes remains limited.

To further elucidate the molecular mechanisms governing apomixis in *E. curvula*, we employed a comparative gene expression analysis to investigate differential gene expression between sexual and apomictic pistils. Using a custom Agilent 1×1M microarray designed from floral transcriptomes, we identified genes whose expression patterns were associated with reproductive mode (Corral et al., 2013; Galla et al., 2017). Microarray hybridization analyses revealed four genes differentially expressed between sexual and apomictic genotypes, suggesting their potential involvement in apomixis regulation, while a fifth candidate gene was identified during genome annotation.

Candidate genes were subsequently validated by assessing their genomic presence across a diverse collection of *E. curvula* genotypes differing in ploidy level and reproductive mode, as well as in a segregating mapping population. Their functional relevance was examined through heterologous expression in *Arabidopsis thaliana*, using ovule-specific promoters and GoldenBraid 2.0-based expression constructs introduced via *Agrobacterium tumefaciens*.

Together, this study integrates comparative gene expression analysis, genomic validation, and heterologous functional assays to identify and characterize candidate regulators of apomixis in *E. curvula*. By providing functional insight into genes associated with reproductive mode determination, our results contribute to the understanding of apomixis regulation and support future efforts aimed at the applied manipulation of clonal seed reproduction in crop species.

## 2 Materials and methods

### 2.1 Microarray design (id 072215)

A customized Agilent array was designed with 1,000 K probes from a floral *E. curvula* reference transcriptome and ESTs previously reported (Cervigni et al., 2008; Garbus et al., 2017). A SurePrint G3 1x1M array format (Agilent) was used. Sequences of 60-mer oligonucleotide probes that were specifically designed (customized) for this study, hereafter referred as CUST, were based on sequences assembled from previously deposited 454 raw read data in the Sequence Reads Archive database (SRA) at NCBI as BioProject 358210 and Expressed Sequence Tag database (dbEST) (N° EH183417 to EH195711). A customized microarray was created using eArray, a free Agilent web-based application that enables the creation of custom microarray designs and oligo libraries (https://earray.chem.agilent.com/earray/). For probe design, the Base Composition Methodology option was employed, and no linker sequences were used. A total of 970,000 probes were obtained from the eArray and were used for the creation of the 1x1M array format. Slides with printed arrays were ordered directly to Agilent Technologies (Santa Clara, CA, USA).

### 2.2 Microdissections and RNA extraction

We grew four sexual and four apomictic *E. curvula* genotypes (diploid and tetraploid individuals; **Table 1**). From each sample, 20–30 florets were collected from developing spikelets at stages determined as described in (Selva et al., 2017). The lemma and palea were removed, and the remaining reproductive organs (pistil and anthers) at equivalent developmental stages were microdissected and pooled. The tissues were manually disrupted in liquid nitrogen using a sterile plastic pestle in 1.5 ml microcentrifuge tubes, and total RNA was extracted using a commercial purification kit (Macherey-Nagel) according to the manufacturer’s instructions. RNA was resuspended in 15 μl of DEPC-treated water and quantified with the RiboGreen^®^ RNA quantitation kit (Molecular Probes, Eugene, OR, USA) using a standard curve. RNA quality was assessed using an RNA 6000 Pico LabChip on the Agilent 2100 Bioanalyzer (Agilent, USA).

**Table 1:** *Eragrostis curvula* genotypes used in the microarray hybridization experiments.

Genotype/ cultivar	Accession (Origin)	Ploidy level	Reproductive mode	Apomictic embryo sacs (%)	References
Tanganyika	PI 234217 (USDA)	4x	Full apomictic	100%	Briggs and Walters, 2016
Don Walter	Don Walter INTA	4x	Facultative	60–100%	Rodrigo et al., 2017
Ermelo	PI 232986 (USDA)	4x	Facultative	80–90%	Zappacosta, 2009
Morpa	PI 591632 (USDA)	4x	Facultative	80–90%	Zappacosta, 2009
OTA-S	PI 574506 (USDA)	4x	Sexual	0%	Zappacosta et al., 2019
920	PI 299920 (USDA)	2x	Sexual	0%	Echenique et al., 1996
214	PI 208214 (USDA)	2x	Sexual	0%	Gallardo et al., 2025
Victoria	UNST1122 (UNS)	2x	Sexual	0%	Cardone et al., 2006

The table lists the genotype, accession and origin, ploidy level, reproduction mode, and percentage of apomictic embryo sacs. The latter represents the proportion of embryo sacs formed through diplospory relative to the total number of embryo sacs observed cytologically. References correspond to published studies documenting the reproductive mode of each genotype. USDA, United States Department of Agriculture; INTA, Instituto Nacional de Tecnología Agropecuaria; UNS, Universidad Nacional del Sur.

### 2.3 *In vitro* transcription and fluorescent labeling

Total RNA extracted from each sample was used as a template for the synthesis of cRNA using the Agilent Low Input Quick Amp Labeling Kit (One-Color) according to the manufacturer’s protocol. Briefly, reverse transcription was carried out to generate first-strand cDNA using an oligo(dT) primer containing a T7 promoter sequence. This was followed by *in vitro* transcription using T7 RNA polymerase in the presence of Cyanine 3 (Cy3)-labeled CTP to generate fluorescently labeled cRNA. The incorporation of Cy3 during transcription enabled one-color detection of gene expression upon hybridization to the microarray. The labeled cRNA was then purified and quantified.

### 2.4 Microarray hybridization procedure

Cy3-labeled cRNA was mixed with 10X Gene Expression Blocking Agent and fragmented using 25X Fragmentation Buffer. The mixture was incubated at 60 °C for 30 min and immediately cooled on ice. An equal volume of 2X Hi-RPM Hybridization Buffer was added to stop the reaction. Gasket slides were put into the chamber base and the mixture was then loaded onto the gasket slides. The gasket slide format was one microarray per slide. Each microarray was carefully set on top of the gasket slide, and then placed on the chamber base. Microarray-gasket assemblies were placed in hybridization chambers, sealed, and hybridized at 65 °C for 17h in a rotating hybridization oven (10 rpm). Following hybridization, microarray slides were washed according to the Agilent Gene Expression Wash Buffer Kit protocol. Finally, the microarray slides were dried by centrifugation at 2,200 rpm for 3 minutes and immediately scanned to minimize signal loss.

### 2.5 Data analysis

Microarray data analysis was performed using the limma package (Linear Models for Microarray Data; R/Bioconductor) (Ritchie et al., 2015). Background correction was performed with the normexp method, followed by between-array normalization, using quantile normalization. Then, a design matrix was constructed to represent the two experimental groups, and linear modeling was applied to each probe. Differential expression (DE) between apomictic and sexual samples was assessed using a contrast matrix specifying the apomictic vs. sexual comparison. Empirical Bayes moderation of standard errors was applied with eBayes to improve variance estimation across CUST. Finally, p-values were adjusted for multiple testing using the Benjamini–Hochberg false discovery rate (FDR) correction. Individual probes from the array were classified as differentially hybridized under p < 0.001; |logFC| >= 2 (Benjamini and Hochberg, 1995).

The probe sequences used in the microarray design, microarray data files, sample metadata and experimental design description, the R script used for DE analysis of the microarray data, the output file containing the results of the analysis, are available at DOI:10.17632/4y7cjz7jyg.1.

### 2.6 Phylogenetic analysis

Based on transcripts identified as differentially hybridized, a phylogenetic tree was constructed to identify potential orthologous genes in close relatives using the Ensembldatabase and the following species: *A. thaliana* (Cheng et al., 2017), *Hordeum vulgare* (Mascher et al., 2021), *Eragrostis tef* (VanBuren et al., 2020), *Zea mays* (Hufford et al., 2021), *Oryza sativa* (Sakai et al., 2013) and different accessions of *E. curvula* (Garbus et al., 2017; Carballo et al., 2019; Selva et al., 2020). First, the transcripts were aligned with BLASTN, and genes with an e-value < 1e-10, Identity >= 70% and coverage >= 70% were retained. The proteins of these genes were aligned using muscle v5.1 (Edgar, 2004) and a maximum Likelihood phylogenetic tree was constructed using MEGA X (Kumar et al., 2018). The topological strength of the trees was evaluated by bootstrap analysis with 1,000 replicates. Finally, the protein domains were detected using CD-search (Marchler-Bauer and Bryant, 2004).

### 2.7 Molecular procedures

To validate results in *E. curvula*, genomic DNA was extracted from fresh leaf tissue following a protocol based on cetyltrimethylammonium bromide (CTAB), as previously reported (Garbus et al., 2017). PCR amplifications were conducted using a MyCycler Biorad™ cycler. Each PCR reaction consisted of 1 μl of 10 mM dNTPs mix, 2.5 μl of 10× reaction buffer, 0.5 μl of each forward and reverse primers (100 pmol/μl), 0.30 μl of DNA polymerase Taq Pegasus^©^ (5 U/µl) and 60 ng of template genomic DNA, in a final reaction volume of 25 μl.

The PCR reaction profile was: initial DNA denaturation at 94°C for 3 min, followed by 40 cycles at 94°C for 30 s, 30 s at the optimal annealing temperature for each primer pair, and 72°C for 30 s. The final step consisted of an extension of 5 min at 72°C. The annealing temperatures for each primer pair were set as the lower melting temperature of both primers. Amplicons were analyzed by electrophoresis in agarose gel (1%) and visualized using ethidium bromide under U.V. light.

### 2.8 Quantitative real-time PCR reactions

The expression of *EcAPO3* and *EcAPO4* in the sexual genotype OTA-S and the apomictic genotype Tanganyika was analyzed by qPCR using a Bio-Rad CFX Connect™ system. Reactions were performed in technical triplicates (15 µL) containing 5 pmol of each primer, five-fold diluted cDNA, and SYBR Green PCR Master Mix (Bio-Rad). Relative expression was calculated using the 2^-ΔΔCt^ method (Livak and Schmittgen, 2001) and normalized with the reference genes *UBICE* and *G6PD* (Selva et al., 2017, Selva et al., 2020). Non-template controls were included. Statistical differences between genotypes were evaluated using a two-tailed Student’s *t*-test based on ΔCt values.

### 2.9 RNA extraction and cDNA first strand synthesis from *E. curvula*

RNA was extracted from approximately 30 mg of *E. curvula* floral tissue using the Promega SV Total RNA Isolation System (Promega). Tissue was homogenized in the kit lysis buffer and centrifuged to pellet cellular debris, and only the cleared supernatant was transferred and applied to the silica membrane spin columns. RNA was bound, washed and treated with RNase-Free DNase I according to the manufacturer’s instructions, and subsequently eluted in Nuclease-Free Water. RNA quantity and quality were assessed with a DeNovix DS-11 spectrophotometer. First-strand cDNA was synthesized using M-MuLV Reverse Transcriptase (NEB) following the manufacturer’s protocol.

### 2.10 DNA and RNA extraction from *A. thaliana*

DNA and RNA were extracted from *A. thaliana* tissues using the TRIzol™ Reagent and procedural guidelines provided by Invitrogen. Briefly, samples were collected in liquid nitrogen and ground immediately using a pestle and mortar. Nearly 100 mg of the powder obtained was transferred to an RNase free 1.5 mL tube and 1 mL of TRIzol™ was added. The aqueous phase containing the RNA was collected and the interphase and organic phase were set-aside for subsequent DNA isolation. The quality and quantity of the nucleic acids were assessed using the DeNovix DS-11 Spectrophotometer. A cDNA first strand synthesis was performed following the same procedure as for *E. curvula*.

### 2.11 Sequence isolation

The Q5 High-Fidelity DNA Polymerase (NEB^©^) was used according to the manufacturer’s instructions to amplify both promoter regions and coding sequences (CDS) by PCR. The 5’ regulatory regions (promoters) of *At5g14010 (KNUCKLES), At2g17950 (WUSCHEL), At4g09960* (*SEEDSTICK*), and *At4g27330* (*SPOROCYTELESS*) were amplified from wild-type *A. thaliana* genomic DNA using specific primers (**Supplementary Table 1**). Gene annotations were retrieved from The Arabidopsis Information Resource (TAIR; *www.arabidopsis.org*). The CDS of *E. curvula*’s candidate genes were obtained from cDNA of the apomictic *E. curvula* cultivar Tanganyika USDA using primers in **Supplementary Table 1**.

### 2.12 Generation of transgenic lines of *A. thaliana*: cloning into expression vectors

To be cloned into GoldenBraid 2.0 (GB2.0) vectors, the amplified sequences were modified at the 5’ end by adding restriction sites for the type IIS enzymes *Esp3I* and *BsaI* (Sarrion-Perdigones et al., 2013). When required, internal restriction sites recognized by those enzymes were removed through the introduction of silent mutations without changing the amino acid sequence, a process referred to as domestication. GB2.0 vectors were used in the subsequent stages of assembly and cloning as previously reported (Sarrion-Perdigones et al., 2013). Firstly, each module containing either a promoter or CDS was cloned in the pUPD2 vector. Afterwards, transcription units (TUs) were generated into pDGBα and pDGB vectors. These cassettes were included in a final expression vector, adding the transcriptional fusion *pNOS:nptII:tNOS* in order to confer kanamycin resistance to transgenic plants.

### 2.13 Bacterial strains and growth conditions

The *Escherichia coli* DH5α strain was used for cloning all GB2.0 vectors, and *Agrobacterium tumefaciens* strain GV3101 was used for stable transformation of *A. thaliana* plants. Both strains were grown in Luria-Bertani (LB) medium under agitation (250 rpm) at 37 °C and 28 °C, respectively. Chloramphenicol (25μg/mL), ampicillin (50 μg/mL), kanamycin (50 μg/mL), or spectinomycin (100 μg/mL) were used for *E. coli* transformant selection. For *A. tumefaciens* transformants selection, colonies were grown in the presence of rifampicin (50 μg/mL) and gentamicin (25 μg/mL). 5-Bromo-4-chloro-3-indolyl-β-D-galactopyranoside acid (40 μg/mL) and isopropylthio-β-galactoside (0.5 mM) were used on LB agar plates for the white/blue selection of clones.

### 2.14 *Arabidopsis thaliana* stable transformation

The *floral dip* method was used to generate transgenic lines of *A. thaliana* (Clough and Bent, 1998). All lines were generated in a Columbia-0 (Col-0) ecotype background. A minimum of 10 lines were obtained for each expression cassette containing the candidate genes. Transgenic plants were grown in plates with Murashige and Skoog (MS) medium and kanamycin (50 μg/mL) as a selective agent (Murashige and Skoog, 1962).

### 2.15 Plant growth conditions

*Arabidopsis thaliana* wild type Col-0 and mutant lines were grown at 22 °C, under 16/8 h day/night photoperiod (with a light intensity of 115 ± 10 μmol s^-1^ m^-2^). The seeds were sterilized in 12.5% (m/v) sodium hypochlorite plus SDS 0,1%, rinsed 4 times in sterile water and plated on MS medium with the appropriate antibiotic. After stratification at 4 °C for 48 h, seeds were transferred to the growth chamber under the conditions described above. Resistant seedlings (3–4 weeks old) were subsequently transplanted to soil.

### 2.16 Morphological and histological analyses

Pistils from different floral developmental stages were dissected and observed under a Leica EZ4 stereomicroscope. All samples were cleared overnight in Hoyer’s solution (Anderson, 1954) and ovules were observed on a Leica DM2500 LED microscope using DIC optics. Images were captured on a Leica MC 170 HD camera using the Leica Application Suite (LAS) EZ software.

## 3 Results

### 3.1 Microarray hybridization and analysis

To perform a comparative gene expression analysis between sexual and apomictic reproductive tissues, we used samples from four tetraploid apomictic and four sexual genotypes (one tetraploid and three diploids) of *E. curvula* (**Table 1**).

A total of eight independent hybridizations were conducted on a custom Agilent microarray platform using a one-color (Cy3-labeled) strategy, with RNA extracted from florets (pistil and anthers) of the selected genotypes, collected at equivalent developmental stages to minimize transcriptional differences unrelated to reproductive mode (**Table 1**).

Microarray hybridization analyses, performed using the custom scripts (**Supplementary Material**), identified 131 differentially hybridized 60-mer sequences between apomictic and sexual samples (p < 0.001; |logFC| >= 2). Notably, 130 of these probes showed higher hybridization signals in apomictic samples, indicating a pronounced transcriptional divergence associated with apomictic development.

### 3.2 Identification and annotation of candidate apomixis-associated genes

BLAST searches of these probes were conducted against the *E. curvula* reference transcriptome database (Garbus et al., 2017) using stringent criteria (100% query coverage and 100% identity). This analysis revealed that 70 probes matched isotig43118, seven aligned with isotig25667, 18 with isotig21895, and two with isotig44764. A subsequent BLASTX search of the identified isotigs against the NCBI nr protein database (accessed Nov, 2024) indicated that these sequences correspond to: a hypothetical protein (designated *EcAPO1*), a pre-mRNA splicing factor CWC22- like homolog (*EcAPO2*), a Cyclin-2A-1 (*EcAPO3*), and an F-box protein (*EcAPO4*) (**Table 2**). Additional probes either aligned to a single isotig or did not meet the minimum identity and coverage thresholds, and were therefore excluded from further analysis. The probe that exhibited downregulation in apomictic genotypes aligned to isotig19617 and corresponds to a hypothetical protein, reported in *E. curvula*.

**Table 2:** BlastX alignment analysis of the four candidate genes against NCBI nr and tsa-nr databases.

Candidate	Reference transcriptome	Protein	Accession	Species	e-value	Identities (%)	Positives (%)	Gaps (%)
*EcAPO1*	isotig43118	Hypothetical protein	JAD67508.1	*Arundo donax*	8e-30	46	59	5
*EcAPO2*	isotig25667	Pre-mRNA-splicing factor CWC22 homolog	PWZ46383	*Zea mays*	0.0	69	78	5
*EcAPO3*	isotig21895	Cyclin-2A-1	XP_006664043	*Oryza brachyantha*	0.0	67	78	1
*EcAPO4*	isotig44764	F-box protein	XP_004962938	*Setaria italica*	3e-77	58	69	6

### 3.3 Analysis of EcAPO candidate genes among genotypes with contrasting reproductive modes

**Figure 1:**
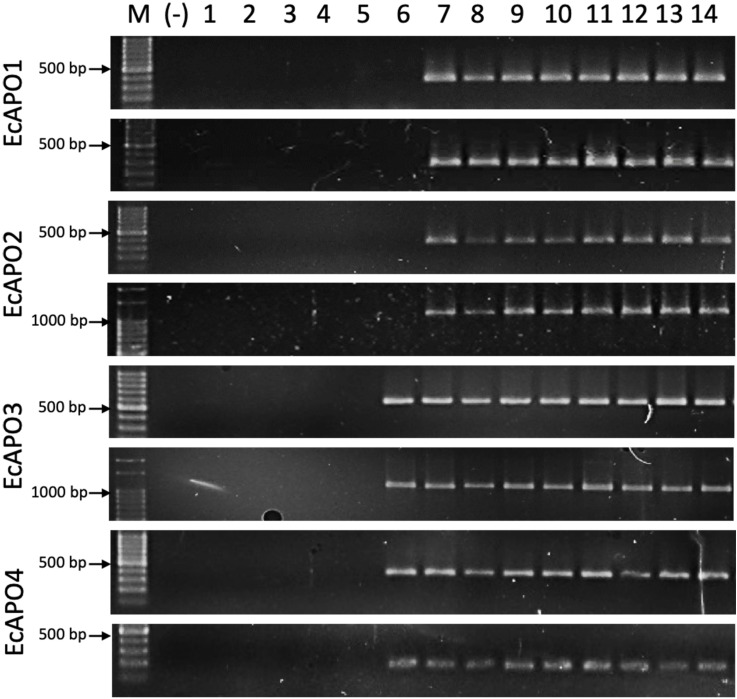
Agarose gel electrophoresis showing the amplification products obtained using genomic DNA of the following *E. curvula* genotypes 1, PI299920; 2, PI208214; 3, PI299919; 4, PI219928; 5, OTA-S; 6, Victoria; 7, Tanganyika; 8, Don Walter; 9, Ermelo; 10, Morpa; 11, Don Pablo; 12, Tanganyika; 13, TUNS9355; 14, Don Luisl; The *EcAPO*-based primer combinations used were: EcAPO1_F1/EcAPO1_R1 and EcAPO1_F2/EcAPO1_R2 for EcAPO1; EcAPO2_F5/EcAPO2_R5 and EcAPO2_F3/EcAPO2_R3 for EcAPO2; EcAPO3_F1/EcAPO3_R1 and EcAPO3_F3/EcAPO3_R3 for EcAPO3; EcAPO4_F1/EcAPO4_R1 and EcAPO4_F2/EcAPO4_R2 for EcAPO4 (**Table 3**). The molecular weight marker consisted of dsDNA fragments ranging from 100 to 1000 bp, evenly spaced at 100 bp intervals, plus two additional bands of 1500 and 3000 bp (PB-L, Argentina). The brighter fragment corresponds to the 500 bp band.

Several PCR primer pairs were designed based on the sequences of the candidate transcripts (**Table 3**) and were tested using the genomic DNA of 14 *E. curvula* genotypes (**Table 4**), including different reproductive modes and ploidy levels. Primer specificity was thoroughly examined using available genomic and transcriptomic resources for *E. curvula*. For all the candidate transcripts, most combinations yielded amplification products of the expected size. Notably, *EcAPO1*- and *EcAPO2-*based primers, produced amplification products exclusively in apomictic genotypes, regardless of ploidy. However, *EcAPO3-* and *EcAPO4*-based primers amplified expected sized fragments not only in the apomictic genotypes, but also in the tetraploid sexual OTA-S (**Figure 1**).

**Table 3:** Primer combinations assayed to amplify the candidate genes.

Primer name	Sequence (5´- 3´)	Primer name	Sequence (5´- 3´)	Estimated fragment size amplified in	
				DNA (bp)	cDNA (bp)
EcAPO1_F1	TCCCTCAACCCTACCGATAA	EcAPO1_R1	AACACCCGTAAGAGCGAATC	~400	N.A.
EcAPO1_F2	TGCTTCGCTCGATGAACTTA	EcAPO1_R2	GCACGGATGTTGCTGATTG	~300	N.A.
EcAPO1_F3	GCGTCTGTGTCGTCTCTTAC	EcAPO1_R3	TTGATCTCGCAGGGTGTTG	~350	~350
EcAPO1_F2	TGCTTCGCTCGATGAACTTA	EcAPO1_R3	TTGATCTCGCAGGGTGTTG	N.A.	~450
EcAPO2_F3	GTCACTGCTGCCGTATAAC	EcAPO2_R3	ACTGCAAGCTCAACACTATC	~1100	N.A.
EcAPO2_F5	GGCATAGGCCAACTTAATGA	EcAPO2_R5	ACATGCACACGCAATACA	~400	N.A.
EcAPO2_F6	ACCAAACCTCCTCTAGCC	EcAPO2_R3	ACTGCAAGCTCAACACTATC	N.A.	~800/~1100
EcAPO2_F1	CTCACCTGGATTGACTGATG	EcAPO2_R6	GTCCATCTCTTGACCAGGATTC	N.A.	~750
EcAPO2_F6	CGTGAGGATGATGGAGAATCTG	EcAPO2_R5	ACATGCACACGCAATACA	N.A.	~800
EcAPO2_F4	ATGGGCTGTTAGCACAAAGG	EcAPO2_R4	TCGAGACGATGCTGTCAAAC	~250	~250
EcAPO3_F1	CAGGTAAGCCACACCTTCATAC	EcAPO3_R1	CGGCACAGGCTACTTCTAATG	~600	~350
EcAPO3_F3	ACCGCAACAATCCAATCAATAAG	EcAPO3_R3	CGAAGGGCTGTGAATGAGAA	~1100	~650
EcAPO3_F1	CAGGTAAGCCACACCTTCATAC	EcAPO3_R2	GGACATGAGAGGCATCCTTATT	N.A.	~650
EcAPO3_qF	TCAGCATCCGCAGTCTTTC	EcAPO3_qR	CCATAAGACGCACACACAAATC	219	125
EcAPO4_F1	GTTCGATTACCTCGTCGTCAA	EcAPO4_R1	CTCCAAGTCTTCCCATCAACA	~350	N.A.
EcAPO4_F2	CTACGCGAACGAGAATGTCTTA	EcAPO4_R2	TTGATGATCCTCCAAGTCTTCC	~200	N.A.
EcAPO4_qF	GCGATCTACTCATCCGAGAAAG	EcAPO4_qR	CCATTGACAAAGACGCTCCTA	~100	~100
EcAPO4_F3	ACTCGCCGCTTCATCAAC	EcAPO4_R2	TTGATGATCCTCCAAGTCTTCC	N.A.	~550

Primers were designed based on the sequences of the candidate transcripts of *Eragrostis curvula*. The predicted amplicon size and the estimated size obtained from DNA and cDNA amplification are shown. N.A.: sequence combinations not assayed.

**Table 4:** *Eragrostis curvula* genotypes assayed to amplify the candidate transcripts indicating seed origin (USDA, United States Department of Agriculture; INTA, Instituto Nacional de Tecnología Agropecuaria; UNS, Universidad Nacional del Sur); ploidy level and reproductive mode.

Sample code	Genotype	Ploidy	Reproductive mode
1	PI299920 USDA	2X	Sexual
2	PI208214 USDA	2X	Sexual
3	PI299919 USDA	2X	Sexual
4	PI219928 USDA	2X	Sexual
5	OTA-S USDA	4X	Sexual
6	Victoria UNS	2X	Sexual
7	Tanganyika USDA	4X	Apomictic
8	Don Walter USDA	4X	Apomictic
9	Ermelo USDA	4X	Apomictic
10	Morpa USDA	4X	Apomictic
11	Don Pablo INTA	7X	Apomictic
12	Tanganyika INTA	4X	Apomictic
13	TUNS9355 UNS	6X	Apomictic
14	Don Luis UNS	6X	Apomictic

### 3.4 Assessing the occurrence of the candidate genes EcAPO1 and EcAPO2 in an *E. curvula* mapping population

**Figure 2:**
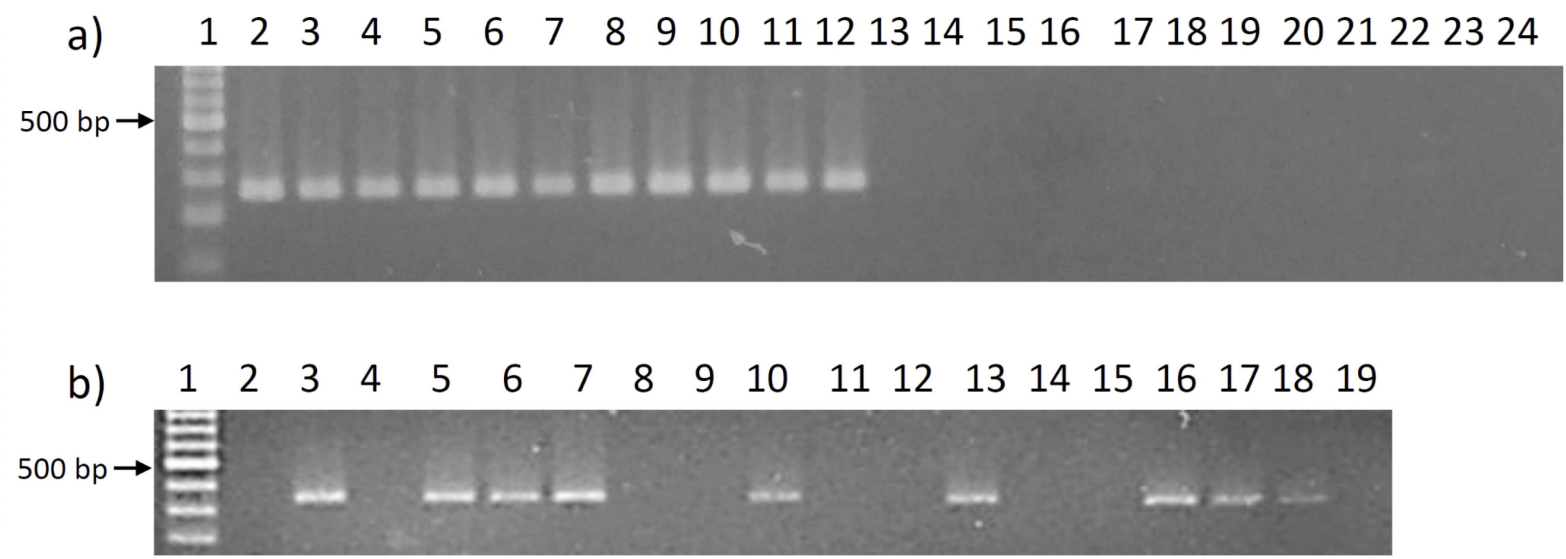
Agarose gel electrophoresis showing the amplification products obtained using genomic DNA from several individuals of the *Eragrostis curvula* mapping population derived from the cross between OTA-S and Don Walter (**Table 5**). **(a)** Amplification using *EcAPO1*-based primers (EcAPO1_F2/EcAPO1_R2) in the genotypes Don Walter (lane 2), apomictic hybrids (lanes 3–12), OTA-S (lane 13) and sexual hybrids (lanes 14–24); **(b)** Amplification using *EcAPO2*-based primers (EcAPO2_F5/EcAPO2_R5) in the genotypes Don Walter (lane 3), OTA-S (lane 4), apomictic hybrids (lanes 5–7, 10, 13, 16-18) and sexual hybrids (lanes 8, 9, 11, 12, 14, and 19). Lane 2 corresponds to the negative PCR control. The molecular weight marker was loaded in lane 1 in both gels and consisted of dsDNA fragments ranging from 100 to 1000 bp at 100 bp intervals, plus two additional bands of 1500 and 3000 bp (PB-L, Argentina). The brighter fragment corresponds to the 500 bp band.

Continuing the inquiry into the potential link between the presence of the candidate genes and apomictic reproduction, *EcAPO1* and *EcAPO2* were tested in a tetraploid mapping population derived from the cross between the fully sexual tetraploid genotype OTA-S and the apomictic tetraploid genotype Don Walter (Zappacosta et al., 2019). Phenotypic characterization of F1 hybrids through cytoembryological analysis resulted in a 1:1 segregation ratio of apomictic to sexual individuals. Consistent with the results obtained in germplasm, PCR amplification products tested using *EcAPO1* and *EcAPO2* specific primers (**Table 3**), were only observed in the hybrids classified cyto-embryologically as apomictic. No amplification was detected in hybrids classified as sexuals (**Figure 2**, **Table 5**). The presence of *EcAPO1* and *EcAPO2* genes in apomictic genotypes, and their absence in sexual ones, suggests that they may play a crucial role in the expression of this trait.

**Table 5:** *Eragrostis curvula* genotypes derived from the mapping population analyzed in **Figure 2**, for: a) *EcAPO1*; and b) *EcAPO2*.

Line	Description	Reproductive mode	Line	Description	Reproductive mode
1	Marker (100bp Plus)		1	Marker (100bp Plus)	
2	Parental	Apomictic	2	Negative	
3	Hybrid02	Apomictic	3	Parental	Apomictic
4	Hybrid08	Apomictic	4	Parental	Sexual
5	Hybrid030	Apomictic	5	Hybrid02	Apomictic
6	Hybrid031	Apomictic	6	Hybrid08	Apomictic
7	Hybrid032	Apomictic	7	Hybrid09	Apomictic
8	Hybrid111	Apomictic	8	Hybrid012	Sexual
9	Hybrid191	Apomictic	9	Hybrid016	Sexual
10	Hybrid194	Apomictic	10	Hybrid017	Apomictic
11	Hybrid197	Apomictic	11	Hybrid020	Sexual
12	Hybrid198	Apomictic	12	Hybrid021	Sexual
13	Parental	Sexual	13	Hybrid024	Apomictic
14	Hybrid025	Sexual	14	Hybrid025	Sexual
15	Hybrid028	Sexual	15	Hybrid028	Sexual
16	Hybrid033	Sexual	16	Hybrid030	Apomictic
17	Hybrid034	Sexual	17	Hybrid031	Apomictic
18	Hybrid039	Sexual	18	Hybrid032	Apomictic
19	Hybrid119	Sexual	19	Hybrid033	Sexual
20	Hybrid128	Sexual			
21	Hybrid140	Sexual			
22	Hybrid158	Sexual			
23	Hybrid174	Sexual			
24	Negative				

### 3.5 Cloning and characterization of the candidate genes

**Figure 3:**
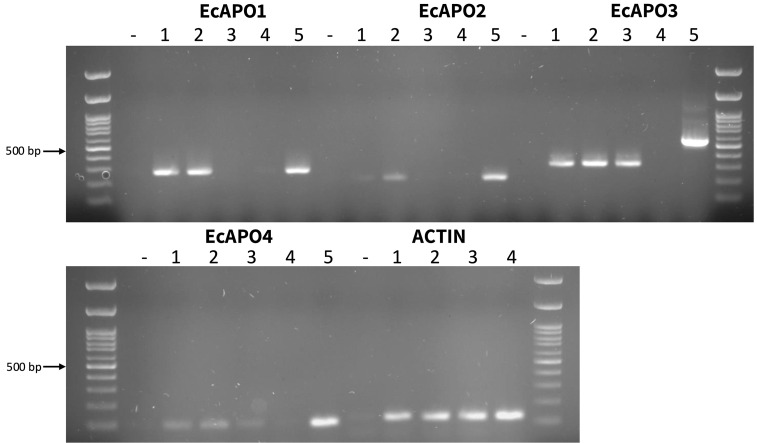
Agarose gel electrophoresis showing the amplification products obtained using cDNA of four *E. curvula* contrasting genotypes: Tanganyika (lane 1), Don Walter (lane 2), OTA-S (lane 3), PI 208214 (lane 4) (**Table 1**), as well as genomic DNA of Don Walter (lane 5) and a negative control for each primer (-). The *EcAPO*-based primer combinations used were: EcAPO1_F3/EcAPO1_R3 for EcAPO1; EcAPO2_F4/EcAPO2_R4 for EcAPO2; EcAPO3_F1/EcAPO3_R1 for EcAPO3; EcAPO4_F1/EcAPO4_qR for EcAPO4. For the housekeeping gene Actin, the assayed primers were 5’-AATGAGCTCCGTGTAGCACCAGAA-3’ (F) and 5’-ACATGGCTGGAACACTGAAGGTCT-3’ (R). The molecular weight marker consisted of dsDNA fragments ranging from 100 to 1000 bp, evenly spaced at 100 bp intervals, plus two additional bands of 1500 and 3000 bp (PB-L, Argentina). The brighter fragment corresponds to the 500 bp band.

The expression of the four candidate genes was analyzed in cDNA synthesized from RNA extracted from inflorescences of the genotypes Tanganyika, Don Walter, OTA-S, and PI 208214 (**Table 1**). As expected, *EcAPO1* and *EcAPO2* were detected exclusively in the apomictic genotypes Tanganyika and Don Walter, whereas *EcAPO3* and *EcAPO4* were also expressed in OTA. None of the genes showed expression in the sexual diploid genotype PI 208214 (**Figure 3**).

Then, selected primer pairs were used to amplify, clone and sequence cDNA corresponding to the candidate genes from the Tanganyika USDA genotype. For *EcAPO1*, a 442 bp fragment was amplified using the primer pair combination EcAPO1_F2/EcAPO1_R3. This sequence was then extended to include both the 5’ and 3’ ends, based on the available transcriptomic and genomic database for *E. curvula*, resulting in a final sequence of 526 bp.

**Figure 4:**
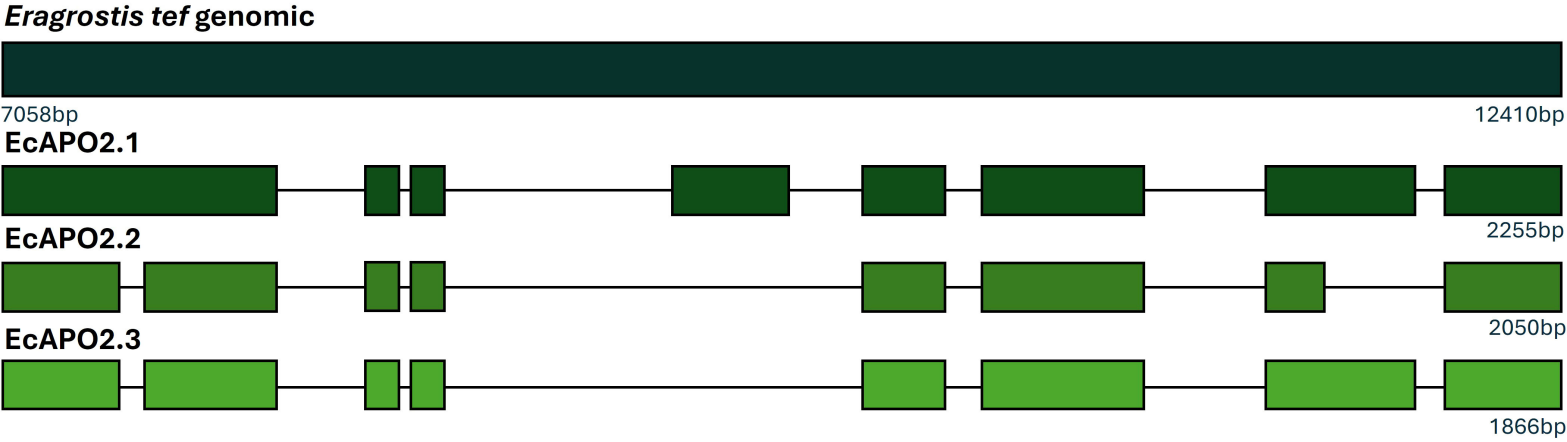
Graphical representation of the alignment (NCBI BLAST) of the three alternative spliced forms of EcAPO2. Amplicons were obtained from Tanganyika USDA cDNA using the primer combinations EcAPO2_F6/EcAPO2_R3, EcAPO2_F1/EcAPO2_R6 and EcAPO2_F6/EcAPO2_R5. The upper sequence represents genomic DNA from *Eragrostis tef*.

In the case of *EcAPO2*, the amplification resulted in three products of different size, suggestive of the expression of three alternatively spliced transcripts. This differential splicing was confirmed through the isolation, cloning and sequencing of these fragments. Given the length of isotig25666, three primer combinations used for that purpose, EcAPO2_F6/EcAPO2_R3, EcAPO2_F1/EcAPO2_R6 and EcAPO2_F6/EcAPO2_R5, yielding fragments of approximately 900, 1000 and 600 bp, respectively. Fragments were cloned, sequenced and assembled, leading to the complete ORF Sequence alignment to *Eragrostis tef* genome, supporting the existence of multiple spliced variants of EcAPO2 (**Figure 4**). Thus, for *EcAPO2* three different spliced transcripts that contained ORF, *EcAPO2_1*, *EcAPO2_2* and *EcAPO2_3*, were detected (**Figure 4**).

*EcAPO3* was amplified using two primer pair combinations, EcAPO3_F1/EcAPO3_R2 and EcAPO3_F3/EcAPO3_R3, both resulting in fragments of approximately 650 bp. The final *EcAPO3* candidate sequence was reconstructed by extending the obtained sequence at both 5’ and 3’ ends, based on the available transcriptomic and genomic database of the species, resulting in a transcript of 1,707 bp.

The cDNA of *EcAPO4* was amplified using the primer combination EcAPO4_F3/EcAPO4_R2, leading to a fragment of approximately 650 bp, that corresponded to the *in silico* predicted F-Box protein, as expected.

To further investigate the relevance of *EcAPO3* and *EcAPO4* in sexual genotypes, their expression levels were analyzed by qPCR. The primers used were EcAPO3_qF/EcAPO3_qR and EcAPO4_qF/EcAPO4_qR for *EcAPO3* and *EcAPO4*, respectively. *EcAPO3* expression did not differ significantly between OTA-S and Tanganyika. However, *EcAPO4* expression was significantly higher in Tanganyika than in OTA-S (p<0.05; **Supplementary Figure 1**).

### 3.6 Phylogenetic analysis

**Figure 5:**
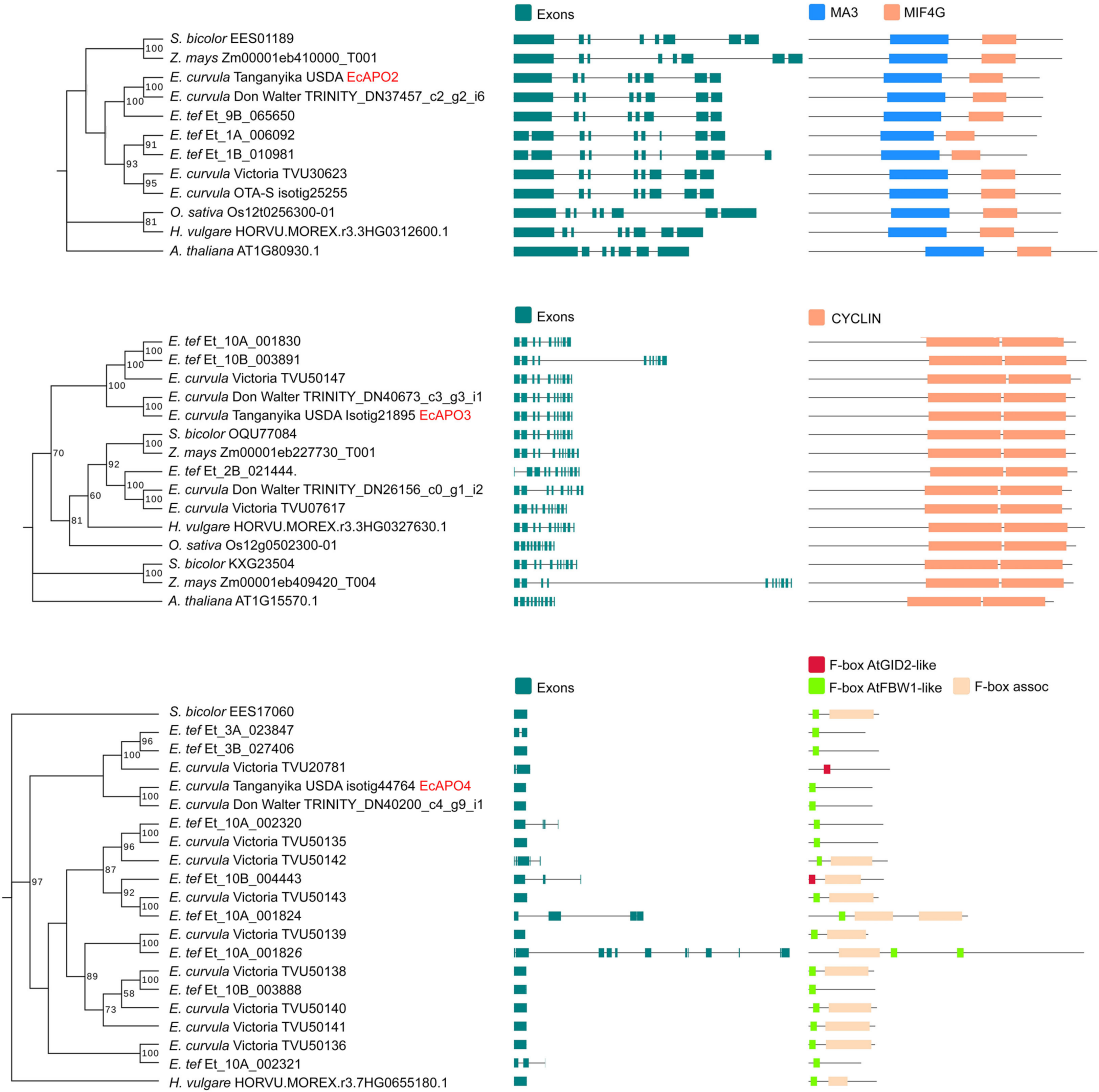
Maximum Likelihood Phylogenetic tree showing the evolutionary relationship of **(A)** EcAPO2, **(B)** EcAPO3 and **(C)** EcAPO4. Evolutionary analysis was performed based on muscle protein alignment. Bootstrap supporting values > 50 are displayed in the nodes. Trees were rooted using the midpoint method.

Orthologous genes were identified for EcAPO2, EcAPO3 and EcAPO4, while they were not found for *EcAPO1*, neither in the selected species nor in the Ensembl database (**Supplementary Table 2**). The *EcAPO2* clade is composed of Tanganyika USDA, Don Walter (TRINITY_DN37457) and the sub-genome 9B of *E. tef* (Et_9B_065650). Even though this gene was also found in other species and genotypes, a speciation event took place in the *EcAPO2* clade. The *EcAPO2* orthologues are well conserved in terms of gene structure and domain since most of them have 8 exons and all of them contain the MA3 and MIF4G domain (**Figure 5A**). *EcAPO3* also showed conserved domains. Two copies of *CYCLYN* were identified within the genes of the analyzed genomes. The gene models have mostly 11 exons, and even though the *E. curvula* is well conserved between genotypes, multiple copies of these genes have been found (**Figure 5B**). *EcAPO4* showed multiple copies in the *Eragrostis* genus. All the proteins contain *F-box* related domains and the gene models have only one exon (**Figure 5C**). For *EcAPO4*, orthologous genes were not found in *A. thaliana* and *Z. mays*.

### 3.7 Engineering of the expression system to trigger apomixis in *Arabidopsis thaliana*

The next goal was to analyze the effects of these candidate apomixis genes when introgressed into a sexual plant, although at this time the transformation of *E. curvula* has been unachievable despite the enormous efforts done by our work group. Hence, the model species *A*. *thaliana* was selected to be transformed through floral dipping mediated through Agrobacterium.

First, *A. thaliana* promoter regions of WUSCHEL, SPOROCYTELESS, SEEDSTICK and KNUCKLES genes (Payne et al., 2004; Bäurle and Laux, 2005; Mizzotti et al., 2014; Mendes et al., 2020) were selected based on their role in ovule development and the establishment of the germline, and then cloned and fused to the candidate genes. The following transcription units (TUs) were generated into pDGBα vectors: *pAtWUS: EcAPO1:tAtACTII; pAtSPL: EcAPO2:tAtUBQ3; pAtSTK: EcAPO3:tNOS; pAtKNU: EcAPO4:t35S and pAtWUS: EcAPO5:tAtACTII*. The TUs were combined by iterative assembling steps alternating between the pDGBα and pDGBΩ vectors (Sarrion-Perdigones et al., 2013) to create the following expression cassettes containing different promoter/candidate gene/terminator combinations, presented in **Table 6**. In addition to the candidates identified via microarray, a fifth gene (*EcAPO5*) was selected during the annotation of the *E. curvula* genome (Carballo et al., 2019), encoding a hypothetical protein, was included in the functional analysis.

**Table 6:** (**A**) Promoters and Terminators used in relation to each *EcAPO* gene analyzed.

(A)					
Promoter	pAtWUS	pAtSPL	pAtSTK	pAtKNU	pAtWUS
*Gene*	*EcAPO1*	*EcAPO2*	*EcAPO3*	*EcAPO4*	*EcAPO5*
*Terminator*	tAtACTII	tAtUBQ3	tNOS	t35S	tAtACTII*

(B)					
	Name of the construct	EcAPO_x1	EcAPO_x3	EcAPO_x4	EcAPO_x5
*Genes*	*EcAPO1*	x	x	x	x
	*EcAPO2*		x	x	x
	*EcAPO3*		x	x	x
	*EcAPO4*			x	x
	*EcAPO5*				x

*promoter/terminator order inverted in the construct.

(B) Names of each of the four designed constructs, marked with a cross are the *EcAPO* genes involved in each one.

Using Agrobacterium and the floral dip method, at least 10 transgenic lines were obtained for each expression cassette into Arabidopsis wild type Col-0 background. All the analyses were conducted on the T1 generation.

### 3.8 Macroscopic phenotyping of transgenic plants

*A. thaliana* has a hermaphrodite flower composed by four concentric whorls: the first whorl (the outer one) consists of 4 sepals, followed by the second whorl with 4 petals, the third whorl with 6 stamens and finally the fourth whorl (the innermost), which contains 2 carpels that develop and fuse to form the pistil.

During reproductive stages, several mutant lines exhibited abnormal phenotypes, including homeotic alterations and indeterminate development. Homeotic phenotypes are the result of alterations related to the genes labeled as homeotic, which are the ones that regulate the precise development of anatomical structures, so they refer to instances where an organ appears in a position where another type of organ is normally found (Bowman et al., 1991).

**Figure 6:**
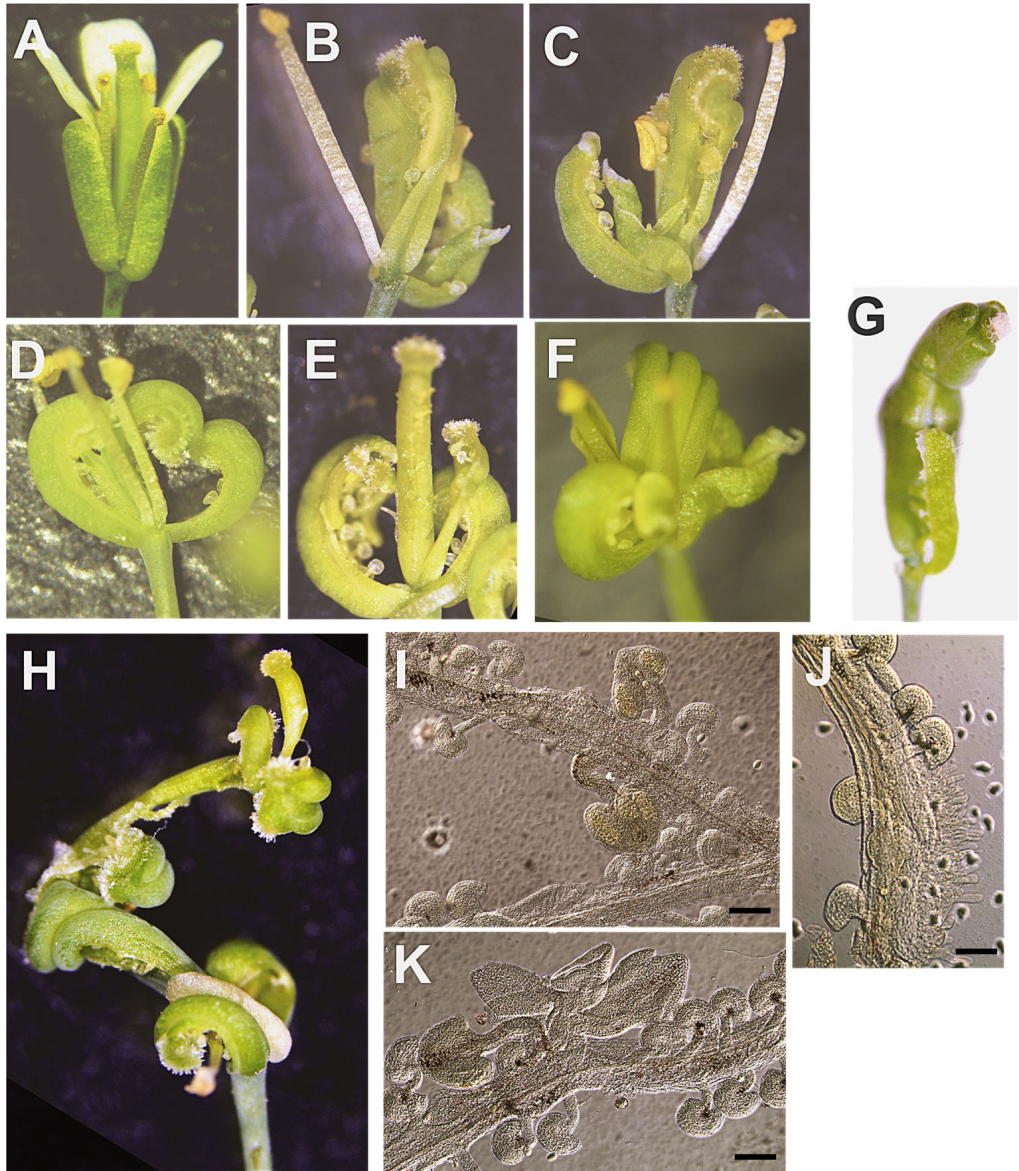
Flower development in WT **(A)** and EcAPO_x5 transgenic plants **(B–K)**, showing homeotic transformations **(B–F)** and indeterminate development **(G–K)**; **(I-K)** pistils observed using DIC microscopy. A 100-µm scale bar is shown.

A total of 10 transgenic lines were analyzed for EcAPO_x3, EcAPO_x4 and EcAPO_x5. The homeotic phenotypes were observed in 60% of the EcAPO_x5 mutants and 30% of EcAPO_x4 mutants, but were absent in EcAPO_x3 (**Figure 6**).

A total of 8 out of the 13 obtained *A. thaliana* mutant EcAPO_x5 lines were analyzed, screening more than 50 plants per line. Carpeloid sepals (10-20%) and flowers with carpeloid sepals and carpel-stamen fusion (80-90%) were observed (**Figures 6B–E**). Only the former were able to produce seeds. Noticeably, no wild type flowers were observed in these mutants. Many flowers exhibited reduced numbers of stamens. In some cases, fusion between carpel and stamen filaments produced rolled pistils and other abnormal developmental patterns (**Figure 6D**). Indeterminate development was also observed, affecting the number of carpels in the innermost whorl, with stamens and/or pistils forming in place of ovules (**Figures 6H–J**).

### 3.9 Microscopic phenotyping of transgenic plants

**Figure 7:**
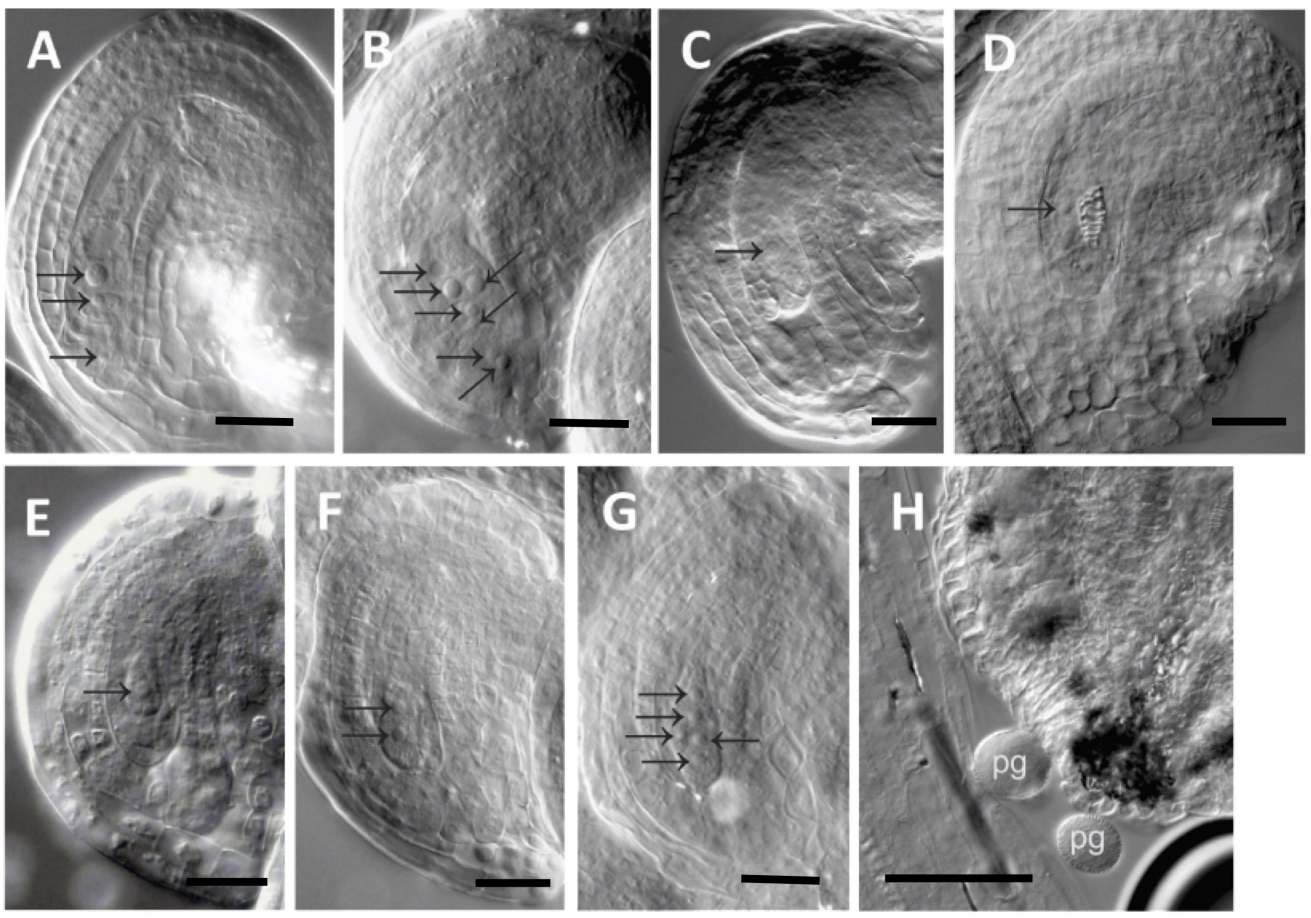
Gametophyte development in EcAPO_x5 mutants. **(A)** mature ES in a WT ovule; **(B)** supernumerary nuclei mutant ovule; **(C)** ES arrest at FG1 stage; **(D)** mutant ES containing cells with somatic identity (tracheids); **(E)** FM in a WT ovule; **(F-G)** mutant ovules showing more than one persistent FM; **(H)** anther of a mutant flower with pollen grains (pg). FM: functional megaspore. A 25-µm scale bar is shown.

Gametophyte development in *EcAPO* transgenic plants was also analyzed using DIC microscopy to identify potential changes related to apomictic development. In *A. thaliana*, the mature ES consists of eight nuclei and seven cells: two gametic cells; the egg and the central cells, along with five accessory cells; two synergids and three antipodal cells (**Figure 7A**). The functional megaspore observed in wild-type ovules is shown in **Figure 7E**.

A total of 381 mutant ovules from EcAPO_x5 transgenic plants and 260 wild-type ovules were analyzed. In the mutant ovules, embryo sacs with supernumerary nuclei were detected, a phenotype not observed in wild-type plants (frequency 0.23 in EcAPO_x5 vs. 0.0 in wild type). These additional nuclei showed atypical positions, mainly affecting the gametic cell region (**Figure 7B**).

During MMC and FM specification, more than one persistent cell was frequently observed at the FM position in mutant ovules (**Figures 7F, G**), a phenotype absent from the wild type (frequency 0.36 vs. 0.0 in wild type). Although the mitotic or meiotic origin of these extra cells remains unknown, their presence could account for the supernumerary nuclei observed at later developmental stages in mature ovules.

In addition, mutant ESs arrested at the FG1 stage or containing cells with somatic identity, including tracheid-like cells, were identified (**Figures 7C, D**). This phenotype also appeared exclusively in the mutants (frequency 0.08 vs. 0.0 in wild type). A deregulation of integument growth was also evident during ovule development, resulting in underdeveloped integuments.

Interestingly, in EcAPO_x4 (1 line, 6 pistils) and EcAPO_x5 lines (2 different lines, average 8 pistils each) exhibiting homeotic phenotypes, early and late abortions ranged between 27 - 67% and 3–6%, respectively. It is important to remark that in Wild Type, those values were 2.7 and 0.6%, respectively, determined by the observation of 330 ovules. Anther structure and pollen grains in mutant flowers are shown in **Figure 7H**.

## 4 Discussion

Understanding the genetic and molecular basis of apomixis is essential not only for advancing knowledge of plant reproductive biology but also for its potential applications in agriculture and plant breeding (Meier et al., 2011; Susmita et al., 2022; Hu et al., 2025). Harnessing apomixis could revolutionize crop improvement by fixing hybrid vigor and ensuring the stable propagation of elite genotypes through seeds, ultimately accelerating breeding cycles and reducing production costs (Hojsgaard, 2020). Despite its crucial importance, the molecular determinants of apomixis remain largely unresolved, particularly in natural systems such as *Eragrostis curvula*, where genome complexity, polyploidy, high heterozygosity and large chromosomal regions with suppressed recombination present major hurdles for gene discovery and genome assembly (Hojsgaard, 2021; Niccolò et al., 2023). This study contributes an integrative approach toward understanding apomixis regulation in *E. curvula*. Through the combination of transcriptomic profiling, molecular validation, and functional assays, we identified and characterized a set of candidate genes associated with reproductive development.

*E. curvula* has been widely used as a model for studying diplosporous apomixis due to the coexistence of sexual and apomictic genotypes within the same species (Cervigni et al., 2008; Briggs and Walters, 2016; Carballo et al., 2019; Pasten et al., 2022). A high-density linkage map has been developed in which the diplospory locus was localized (Briggs and Walters, 2016; Zappacosta et al., 2019). Complementary studies identified differential methylation patterns, small RNA populations, and expression changes between apomictic and sexual genotypes, revealing that apomixis is a polygenic and epigenetically modulated process governed by a complex regulatory network controlling reproductive fate (Rodrigo et al., 2017; Garbus et al., 2017; Zappacosta et al., 2019; Selva et al., 2020; Pasten et al., 2022). Despite these advances, a comprehensive functional analysis is still required to fully understand the specific mechanisms of apomixis in this and other natural systems.

Microarray technology has proven to be efficient for the identification of differentially expressed genes between contrasting genotypes, overcoming challenges associated with genome complexity (Amiteye et al., 2011; Amiteye et al., 2013; Ong-Abdullah et al., 2015; Yazdani et al., 2023).

In this work, custom made microarrays were hybridized with genotypes contrasting in both reproductive mode (sexual vs. apomictic) and ploidy (diploid vs. polyploid), dissecting transcriptional differences between sexual and apomictic *E. curvula* genotypes. The custom Agilent platform enabled the detection of 131 differentially hybridized sequences, among which four (*EcAPO1*–*EcAPO4*) showed the strongest association with apomictic reproduction. These genes encoded a hypothetical protein (*EcAPO1*), a pre-mRNA splicing factor CWC22 -like homolog (*EcAPO2*), a *Cyclin-2A-1* (*EcAPO3*), and an F-box protein (*EcAPO4*). Notably, genomic copies of *EcAPO1* and *EcAPO2* were present exclusively in apomictic genotypes either in the germplasm collection and in a segregating mapping population supporting their close linkage with apomixis. In contrast, *EcAPO3* and *EcAPO4* were also detected in the sexual tetraploid OTA-S Interestingly, although *EcAPO4* was present in both sexual and apomictic genotypes, its higher expression in Tanganyika suggests that differential gene expression, rather than gene presence alone, may contribute to the regulation of apomictic development in *E. curvula* and could also reflect a broader functional role related to ploidy or general developmental regulation.

Alternative splicing of *EcAPO2*, resulting in three isoforms, highlights an additional regulatory layer. Moreover, *EcAPO2* appears to have undergone a speciation event in *E. curvula* apomictic genotypes and exclusively within the 9B subgenome of *E. tef*. In the Victoria and OTA-S genotypes, the homologous *EcAPO2* gene retains its structural and domain organization; however, its sequence shows considerable divergence with the apomictic one, exhibiting only 67.4% protein identity. Additionally, a partial fragment of Isotig25255, and therefore of the predicted protein TVU30623, were detected in the Don Walter transcriptomes, suggesting that this cultivar likely possesses two distantly related *EcAPO2* homologs. Future studies using isoform-specific quantitative approaches will be necessary to determine whether the different *EcAPO2* splice variants show differential expression patterns or functional specialization during apomictic development in *E. curvula*.

Concerning the presence of *EcAPO3* and *EcAPO4* in the tetraploid sexual genotype OTA-S, it is likely that their presence in OTA-S reflects a ploidy-related retention of certain genomic regions, without direct involvement in reproductive fate determination. Alternatively, *EcAPO3* and *EcAPO4* might act as general regulators, with their role in apomixis specifically emerging only in the presence of other interacting factors absent in strictly sexual genotypes. *EcAPO3* is highly conserved and contains two cyclin domains across all the analyzed sequences. *EcAPO4* phylogenetic analysis showed that the multiple duplication events arose in the *Eragrostis* clade while in other species such as *Z. mays* and *A. thaliana* no orthologous genes were found. *EcAPO4* speciation was found in the apomictic Tanganyika USDA and Don Walter cvs. F-box proteins are one of the biggest families in plant species and are usually related with ubiquitin ligases (Xu et al., 2009). In this way, *EcAPO4*, which contains an F-box domain, could be related with the discovery of differentially expressed genes involved in the ubiquitin pathway previously linked to apomixis in *E. curvula* (Selva et al., 2020). For *EcAPO2*, *EcAPO3* and *EcAPO4* a speciation event was found in the apomictic genotypes.

Functional validation of these genes in *E. curvula* has been hindered by the unsuccessful efforts to develop a transformation system for this species, and thus constituting a persistent constraint for apomixis disclosure (Bellido et al., 2021). To overcome this, we employed *Arabidopsis thaliana* as a heterologous model to test the effects of introducing *EcAPO* genes under A. *thaliana* promoters.

Concerning the promoters expression, WUSCHEL is active in the organizing center of the shoot apical meristem (SAM), where it maintains the pool of undifferentiated stem cells (Mayer et al., 1998). During early embryogenesis, it is also expressed beneath the embryonic shoot meristem, and in floral meristems it becomes restricted to the basal region. The KNUCKLES promoter becomes active during late floral development. It is induced around floral stage 6 under the control of AGAMOUS (Payne et al., 2004). Its expression begins in emerging carpel primordia and later appears in developing stamens and ovules. Overall, KNU expression in the floral meristem’s central zone represses WUS to terminate stem cell maintenance (Sun et al., 2009; Kwaśniewska et al., 2021). The SPOROCYTELESS promoter is active in the sporogenous precursor cells of both anthers and ovules. Since it is expressed in the microsporocytes of anthers and in the nucellus region of ovules during sporogenesis, it functions during both male and female spore formation (Yang et al., 1999). Finally, the SEEDSTICK promoter drives expression in the female reproductive organs, especially the gynoecium and developing ovules. *In situ* hybridization assays demonstrated activity in carpels, placenta tissue, and ovule primordia, consistent with its role in ovule identity and seed coat development (Pinyopich et al., 2003).

The obtained transgenic lines displayed a range of abnormal floral and reproductive phenotypes, including homeotic transformations, developmental arrests, supernumerary nuclei, and abnormal embryo sac differentiation. These alterations suggest that the *EcAPO* genes are capable of interfering with conserved regulatory pathways governing reproductive development, and the evidence observed supports the hypothesis that their native role in *E. curvula* may involve the modulation of cell fate decisions at early reproductive stages.

EcAPO_x5 and EcAPO_x4 mutants exhibited striking homeotic transformations, including sepal-to-carpel conversions and fusion of stamens and carpels into chimeric structures, whereas these were not detected in the mutants EcAPO_x3 and EcAPO_x1. These phenotypes are reminiscent of mutants in *Arabidopsis thaliana*, such as *apetala2 (ap2), apetala3 (ap3), and superman (sup)* (Bowman et al., 1991). In *ap2* mutants, sepals are transformed into carpeloid organs sometimes bearing ovules and stigmatic tissue at their margins, accompanied by reduced stamen numbers and incompletely fused carpels (Bowman et al., 1991; Jofuku et al., 1994; Huang and Ma, 1997). Similarly, ectopic or constitutive expression of the C-class gene *AGAMOUS* (AG) or its homologs can replace sepals with carpeloid structures (Rutledge et al., 1998; Rigola et al., 2001; Boss et al., 2001; Li et al., 2002). Moreover, hypomethylated backgrounds have been found to produce carpeloid sepals due to misexpression of AG and APETALA3 (AP3) (Finnegan et al., 1996; Madlung et al., 2002). In this hypomethylated background the gene SUPERMAN (SUP) was observed to be hypermethylated (Jacobsen and Meyerowitz, 1997).

The fusion of stamens and carpels observed in *EcAPO* mutant plants parallels the phenotypes observed in *sup* mutants, particularly the so-called “*superwoman*” class, which exhibit indeterminate floral meristems with increased numbers of incompletely or completely fused carpels, forming multilocular pistils (Breuil-Broyer et al., 2016). *SUP* normally restricts B-class gene activity to whorls 2 and 3, preventing ectopic AP3/PI function in carpels. Loss of *SUP* function or altered epigenetic regulation of SUP can thus generate pistillody and stamen-carpel chimeras (Bowman et al., 1992; Goto and Meyerowitz, 1994). Indeed, *SUP* allele mutants such as *sup-epiA31* or *fon1* produce chimeric stamen-carpel organs, and double mutants (*ap2 fon1, clv fon1*) display additive phenotypes, combining carpeloid sepals with chimeric stamens (Jacobsen and Meyerowitz, 1997; Huang and Ma, 1997). The phenotypic resemblance to *sup* mutants suggests that *EcAPO* genes may be interfering with pathways regulating floral meristem determinacy, potentially involving epigenetic components (Jacobsen and Meyerowitz, 1997).

Interestingly, in oil palm, severe homeotic transformations resulting in staminodes and stamens developing as pseudocarpels were observed. These abnormalities are caused by hypomethylation of the KARMA transposon, an ortholog of AP3, which results in an alternatively spliced transcript that leads to a truncated peptide (Ong-Abdullah et al., 2015). The role of the spliced forms of the EcAPO2 gene needs to be investigated, as both results suggest regulatory plasticity in pre-mRNA processing as a component for the modulation of reproductive pathways.

At the reproductive level, the effects of the *EcAPO* genes extended to ovule development. Normally, the megaspore mother cell undergoes meiosis to produce a functional megaspore, giving rise to an eight-nucleate embryo sac (Yang et al., 2010). Introgression of *EcAPO* genes into *A. thaliana* induced phenotypes that resemble certain cellular features associated with diplosporous development, such as supernumerary FM-like cells.

Taken together, our results identify novel candidate genes associated with apomixis in *Eragrostis curvula* and provide functional evidence supporting their involvement in reproductive development. By integrating comparative gene expression profiling, genomic association analyses, and heterologous functional assays, this study highlights the regulatory complexity underlying apomixis and reinforces the view of this trait as a multifactorial and developmentally regulated process rather than a single-gene phenomenon. Although functional validation in the native system remains technically challenging, the biological activity observed in *Arabidopsis thaliana* underscores the relevance of these candidates to conserved reproductive pathways. Future studies aimed at dissecting their molecular interactions, epigenetic regulation, and downstream targets will be essential for advancing our understanding of apomixis and for informing long-term efforts toward the engineering of clonal seed reproduction in crop species.

## Data Availability

The Transcriptome Shotgun Assembly project has been deposited at DDBJ/EMBL/GenBank under the accession GFVM00000000, as reported in Garbus et al. (2017). ESTs are deposited in GenBank (EH183417 to EH195711), as reported in Cervigni et al. (2008). Sequence data related to the candidate genes are included in the International Patent WO2025114950A1 – ‘Nucleic acid constructs comprising apomictic genes of Eragrostis curvula and methods related thereto’.

## Supplementary material

Supplementary Figure 1 Relative expression levels of EcAPO3 and EcAPO4 in the sexual genotype OTA-S and the apomictic genotype Tanganyika. Gene expression was quantified by qPCR and normalized using the reference genes UBICE and G6PD. Expression levels were calculated using the 2^−ΔΔCt method. Bars represent mean ± SD. Asterisks indicate statistically significant differences between genotypes (*p* < 0.01; two-tailed Student’s *t*-test).
