# Supplementary material for: Integrative gene expression and heterologous functional analysis identify candidate regulators of apomixis in Eragrostis curvula
Source: Front Plant Sci. 2026 Apr 15;17:1802327. doi: 10.3389/fpls.2026.1802327 (PMC13126312; doi:10.3389/fpls.2026.1802327)
Supplement: Supplementary file 1 [file Table1.docx]

| Table S1. Primers used during GB2 cloning. | |
| --- | --- |
| pSPLplusFw1 | GCTCGTCTCTCTCGGGAGTGCTTTCG |
| pSPLplusRv1 | GCTCGTCTCTAGTCAAGtGACGTTGAAAAAAATGC |
| pSPLplusFw2 | CGTCGTCTCTGACTTACACCCACTAATATTGAC |
| pSPLplusRv2 | GCTCGTCTCTCTCAATGGTGATGATGATCTTC |
| pKNUplusFw | GCTCGTCTCTCTCGGGAGTTGTGTGTG |
| pKNUplusRv | GCTCGTCTCGCTCAATGGGAGAGGTTCTTAAGC |
| pWUSplusFw | GCTCGTCTCGCTCGGGAGAGTTTGGTGAC |
| pWUSplusRv | GCTCGTCTCTCTCAATGGGTGTGTTTGATTC |
| pSTKplusFw | GCTCGTCTCACTCGGGAGCCAACGA |
| pSTKplusRv | GCTCGTCTCGCTCAATGGTCTGGAGAGAC |
| HP1.GB2.Fw | GCTCGTCTCTCTCGCCATCGTAACCCTAGTCCCAAG |
| HP1.GB2.Rv | GCTCGTCTCTCTCAAAGCAGCCTGACAAGCACAGAT |
| HP2.GB2_Fw | GCTCGTCTCTCTCGCCATCGCCCTCTTCTCTCCATG |
| HP2.GB2_Rv | GCTCGTCTCTCTCAAAGCACGCATACAGAACGCTAC |
| SPs.GB2.Fw | GCTCCGTCTCCCTCGCCATACCAAACCTCCTCTAGCC |
| SPs.GB2.Rv | GCTACGTCTCTCTCAAAGCTCAGAACTCACGAGATCCT |
| CIC.GB2.Fw | GCTCGTCTCTCTCGCCATTCGATTTCAGGCTTACATGG |
| CIC.GB2.Rv | GCTCGTCTCTCTCAAAGCGGTATCATCATCAGCTCGT |
| FBOX.GB2.Fw1 | GCTCGTCTCTCTCGCCATGATGGACAAGCTCACCG |
| FBOX.GB2.Fw2 | GCTCGTCTCCACCTGtGTCTCCAGGCACTG |
| FBOX.GB2.Rv1 | GCTCGTCTCCAGGTaAAGCGtCGGAGGGACCT |
| FBOX.GB2.Fw3 | GCTCGTCTCAaGCaaGaCTTGGtTTCGACCCTGCTGTCTCCTCTCGCTTCCATG |
| FBOX.GB2.Rv2 | GCTCGTCTCTtGCtGTCTCCACtTTGTTGGAAACCCACG |
| FBOX.GB2.Rv3 | GCTCGTCTCTCTCAAAGCCGTGCTGTGCTA |
