# Supplementary figures and images for: Integrative gene expression and heterologous functional analysis identify candidate regulators of apomixis in Eragrostis curvula
Source: Front Plant Sci. 2026 Apr 15;17:1802327. doi: 10.3389/fpls.2026.1802327 (PMC13126312; doi:10.3389/fpls.2026.1802327)

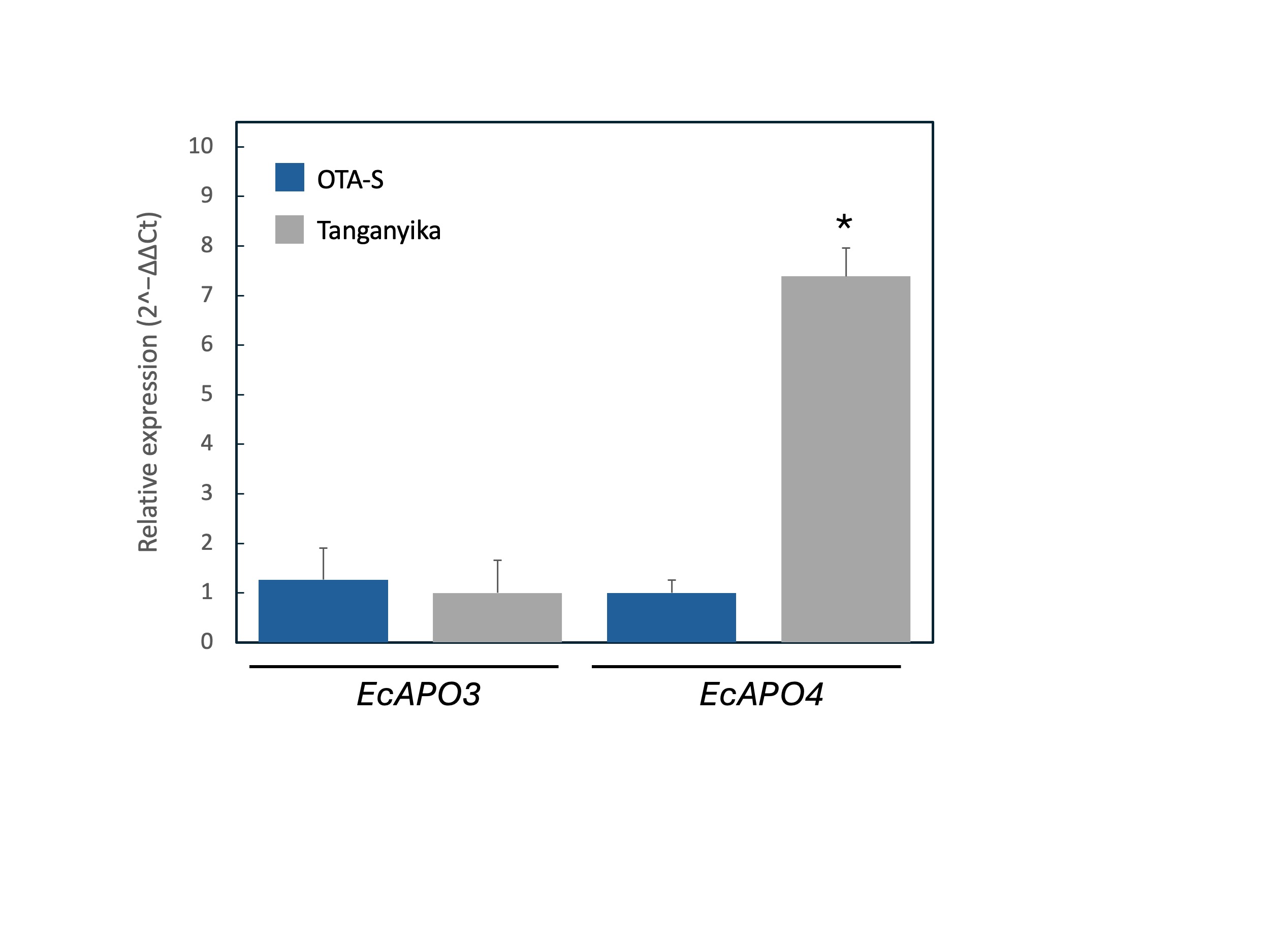

Supplement: Supplementary Figure 1 — Relative expression levels of EcAPO3 and EcAPO4 in the sexual genotype OTA-S and the apomictic genotype Tanganyika. Gene expression was quantified by qPCR and normalized using the reference genes UBICE and G6PD. Expression levels were calculated using the 2^−ΔΔCt method. Bars represent mean ± SD. Asterisks indicate statistically significant differences between genotypes (p < 0.01; two-tailed Student’s t-test). [file Image1.jpeg]
